# A comparative analysis of genes differentially expressed between rete testis cells and Sertoli cells of the mouse testis

**DOI:** 10.1038/s41598-023-48149-7

**Published:** 2023-11-28

**Authors:** Ekaterina A. Malolina, Adelya A. Galiakberova, Valery V. Mun, Marat S. Sabirov, Erdem B. Dashinimaev, Andrey Yu. Kulibin

**Affiliations:** 1 Koltzov Institute of Developmental Biology, Russian Academy of Sciences, 119334 Moscow, Russia; 2 Center for Precision Genome Editing and Genetic Technologies for Biomedicine, Pirogov Russian National Research Medical University, 117997 Moscow, Russia; 3 Moscow Institute of Physics and Technology (State University), Institutskiy Per., 141701 Dolgoprudny, Russia

**Keywords:** Differentiation, Testis, Gene expression analysis, Reproductive biology

## Abstract

The rete testis (RT) is a region of the mammalian testis that plays an important role in testicular physiology. The RT epithelium consists of cells sharing some well-known gene markers with supporting Sertoli cells (SCs). However, little is known about the differences in gene expression between these two cell populations. Here, we used fluorescence-activated cell sorting (FACS) to obtain pure cultures of neonatal RT cells and SCs and identified differentially expressed genes (DEGs) between these cell types using RNA sequencing (RNA-seq). We then compared our data with the RNA-seq data of other studies that examined RT cells and SCs of mice of different ages and generated a list of DEGs permanently upregulated in RT cells throughout testis development and in culture, which included 86 genes, and a list of 79 DEGs permanently upregulated in SCs. The analysis of studies on DMRT1 function revealed that nearly half of the permanent DEGs could be regulated by this SC upregulated transcription factor. We suggest that useful cell lineage markers and candidate genes for the specification of both RT cells and SCs may be present among these permanent DEGs.

## Introduction

The mammalian testis consists of seminiferous tubules where SCs support the production of spermatozoa from undifferentiated germ cells, the interstitium with testosterone-producing Leydig cells, and the RT. The RT is a system of interconnected cavities and channels through which spermatozoa are transported out of the testis.

Recent studies on mice have demonstrated that the RT strongly influences the formation of the Sertoli valve, a transition region which is located at the border between seminiferous tubules and the RT and composed of SCs and undifferentiated spermatogonia^1,2^. The Sertoli valve prevents a detrimental backflow of the RT fluid into seminiferous tubules^2^. In addition, SCs in the transition region exhibit an immature phenotype and prolonged proliferation in hamsters^3^ and rats^4,5^ which also might be influenced by the RT. It has been reported that seminiferous tubules also may affect RT function by luminal factors^6^.

The importance of the RT for the whole testis physiology suggests the need for further research on this region. The origin of the RT epithelium is not completely studied. Morphological, immunohistochemical^7–10^ and single-cell RNA-seq (scRNA-seq) data^11^ indicate that mouse RT cells develop from the same gonadal precursor cells as SCs. However, the timing of their specification, the relationship between RT cells and SCs, as well as the presence of different subpopulations inside the RT epithelium are still a matter of debate^10,11^. Defining differences in gene expression between RT cells and SCs could be useful for addressing these issues.

Previously we performed bulk RNA-seq of adult RT cells in culture and identified genes specific to the adult mouse RT^12^. However, the culture was admixed with peritubular myoid cells which hindered the data interpretation. Another study utilized a microarray to study the transcriptome of the adult RT in vivo but it was also not free from admixtures^1^. Finally, scRNA-seq of the fetal RT^11^ and the juvenile RT^2^ was performed. However, there were no reports on RNA-seq of the RT from neonatal mice. In the current study, we filled this gap and conducted bulk RNA-seq of RT cells and SCs from neonatal (5–6 dpp, days postpartum) mice. For that purpose, we took advantage of a cell sorting strategy and obtained pure cultures of both SCs and cells from the RT epithelium.

After analyzing DEGs between neonatal RT cells and SCs, we performed a comparative analysis to reveal genes that are permanently differentially expressed between these two cell populations throughout testis development and in culture. We suggest that useful cell lineage markers and candidate genes for the specification of both RT cells and SCs may be among such genes. To achieve this aim, we compared DEGs from our study with DEGs obtained after analyzing fetal^11^ and juvenile^2^ testicular cells. In this way, we identified SC- and RT-specific genes that are common to fetal and postnatal ages and also to in vivo and in vitro conditions. We demonstrated that a number of these genes are differentially expressed between adult RT cells and adult SCs as well.

## Results

### Establishment of a pure culture of RT cells by FACS

Pure populations of neonatal (5–6 dpp) RT cells and SCs were needed to accurately compare their transcriptomes using bulk RNA-seq. Pure SCs were obtained by the method developed in our previous study which was based on labeling peritubular myoid cells with PDGFRA antibody and then sorting them out of the cell suspension of seminiferous tubules by FACS^13^. PDGFRA^-^ cell fraction was cultured for 3 days to eliminate germ cells. As a result, SCs identified by WT1 staining accounted for 93.3 ± 2.4% of cells in the culture (Supplementary Fig. S1).

**Figure 1:**
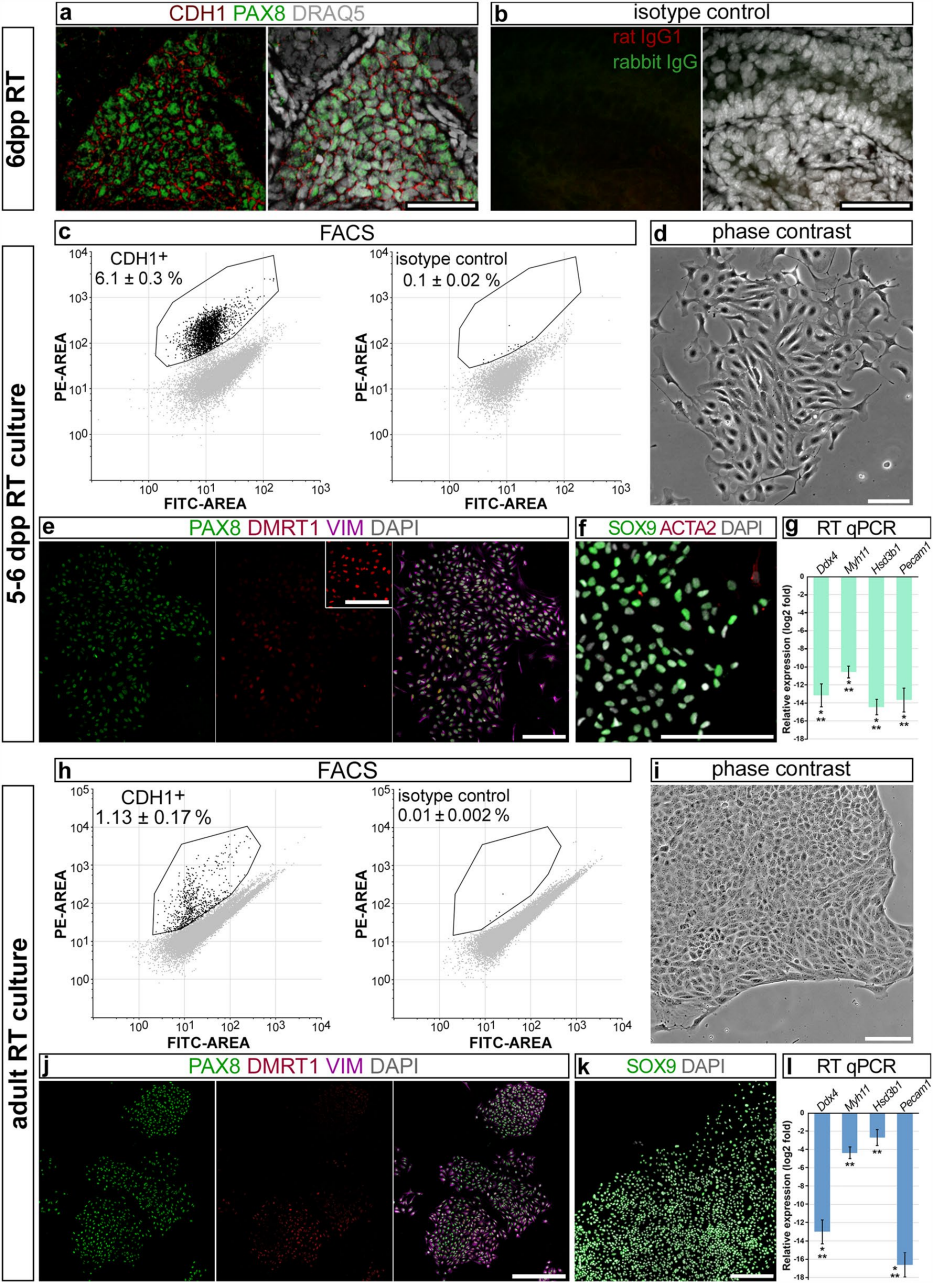
Characterization of RT cell cultures. (**a**) Whole-mount immunofluorescent staining of the neonatal RT with PAX8 antibody and CDH1 antibody for flow cytometry. (**b**) Staining with isotype control antibodies exhibited no specific signal. (**c,h**) FACS of cells isolated from RT regions of neonatal (**c**) and adult mice (**h**). The cells were stained with CDH1 and isotype control antibodies labeled with PE. Debris and doublets were excluded from the analysis. The percentage of CDH1 + cells was presented as the mean ± SEM from three independent experiments. (**d,i**) Morphological appearance of neonatal (**d**) and adult (**i**) RT cell cultures on day 3 and day 8 respectively. (**e,j**) Triple immunofluorescent staining of neonatal (**e**) and adult (**j**) RT cell cultures for PAX8, DMRT1, and vimentin (Vim). The inset in (**e**) shows DMRT1 staining of a neonatal SC culture for comparison. (**f,k**) Immunofluorescent staining of neonatal (**f**) and adult (**k**) RT cultures for SOX9; the neonatal culture (**f**) was co-stained for ACTA2. Images in (**e**,**f**,**j**,**k**) were stitched from several adjacent fields of view. (**g,l**) mRNA levels of markers of different testicular cell populations in neonatal (**g**) and adult (**l**) RT cultures relative to that in adult testicular tissue (the zero line). Data are presented as the mean ± SEM from three independent experiments. **p < 0.01, ***p < 0.001. Scale bars: 50 μm (**a**,**b**), 100 μm (**d–f**,**i**–**k**).

To obtain a pure culture of RT epithelial cells we also applied FACS. It was shown in the study of Nagasawa et al.^14^ and also in our previous study^12^ that all RT cells expressed surface marker cadherin 1 (CDH1). So, we first verified that a CDH1 antibody for flow cytometry stained cells of the RT (Fig. 1a,b). Next, we used this antibody to sort CDH1 + cell fraction from a neonatal testicular cell suspension (Fig. 1c). The cells were maintained in culture for 3 days to eliminate spermatogonial cells which also expressed CDH1^15^.

As we previously described^12^, RT cells formed colonies in culture (Fig. 1d), which were positively stained for PAX8 (Fig. 1e) and SOX9 (Fig. 1f). The number of PAX8^+^ cells was 93.4 ± 0.8%. Some RT cells also expressed DMRT1 (Fig. 1e). SCs identified as PAX8^-^DMRT1^+^ cells were practically not observed in the culture whereas peritubular myoid cells identified by ACTA2 staining were present in a small number (Fig. 1f). All cells in the culture expressed somatic marker vimentin (Fig. 1e), and there were no cells positive for germ cell marker DDX4 (Supplementary Fig. S2). Real-time qPCR (RT qPCR) analysis showed that transcript levels of markers of germ (*Ddx4*), myoid (*Myh11*), Leydig (*Hsd3b1*), and endothelial cells (*Pecam1*) were low compared with those in testicular tissue (Fig. 1g).

We also demonstrated that such an isolation procedure could be applied to adult RT cells. After cell staining and FACS (Fig. 1h), CDH1^+^ cell fraction was cultured for 8 days. RT cells formed large colonies (Fig. 1i) stained for PAX8 (Fig. 1j) and SOX9 (Fig. 1k), DMRT1 was present in some cells (Fig. 1j). The number of RT cells was 94.1 ± 1.5% according to PAX8 staining, while the number of SCs was negligible. All cells in the culture were positive for vimentin (Fig. 1j) and negative for DDX4 (Supplementary Fig. S2). RT qPCR data confirmed the purity of the culture (Fig. 1l).

### Identification of DEGs between neonatal RT cells and SCs and their functional analysis

**Figure 2:**
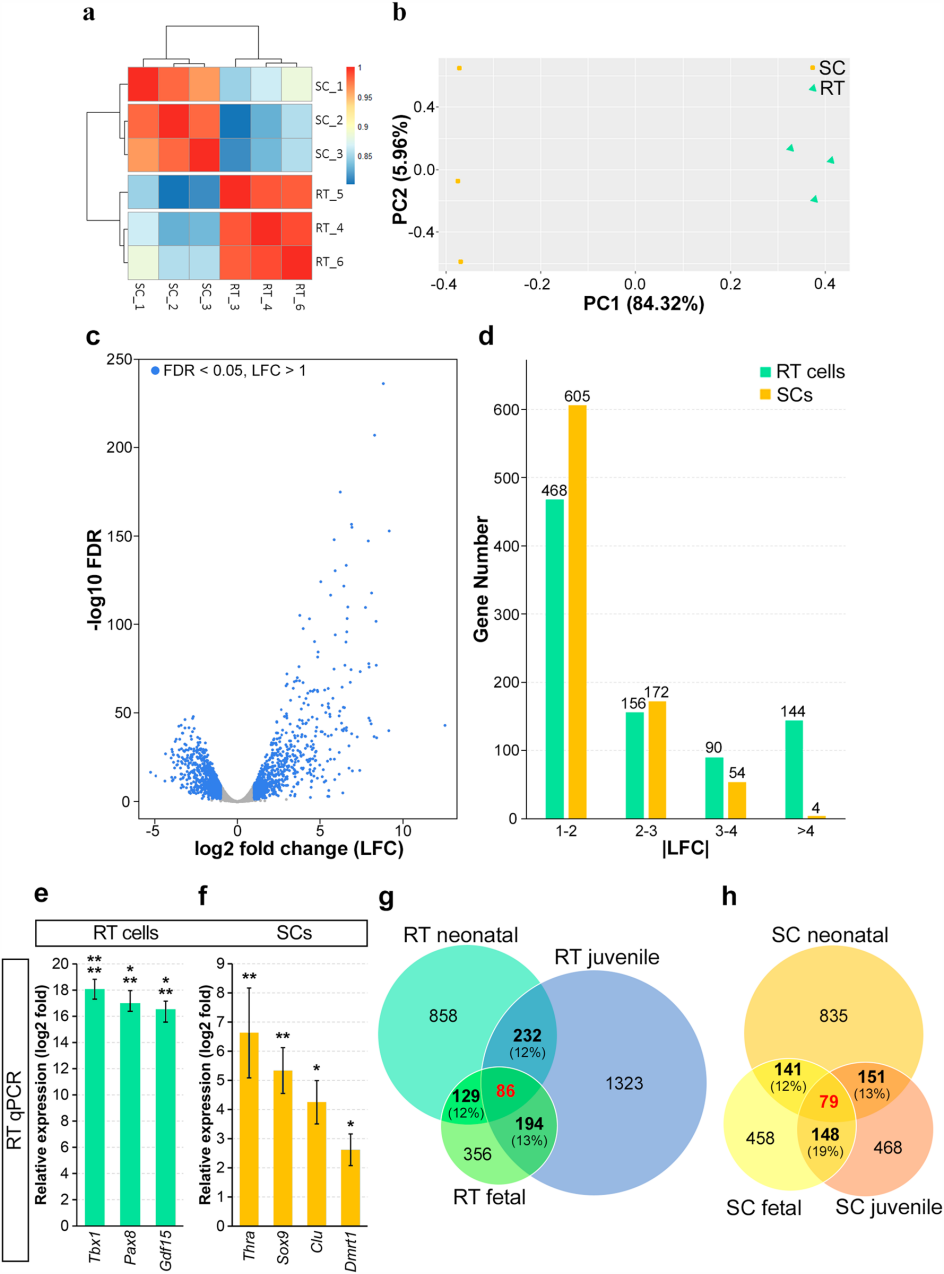
RNA-seq analysis of cultures of neonatal RT cells and SCs with the comparison with the data of the other studies. (**a**) A heatmap of Pearson correlation coefficients between RT and SC samples. (**b**) A plot of PCA analysis showing a cluster of RT cells and a cluster of SCs. (**c**) A volcano plot of the RNA-seq data. DEGs selected for the further analysis are highlighted in blue. (**d**) A histogram of LFC absolute values for genes differentially expressed between RT cells and SCs. (**e**,**f**) Validation of RNA-seq data by RT qPCR in cultures of neonatal RT cells (**e**) and SCs (**f**); the zero line represents mRNA levels in an SC culture (for **e**) and mRNA levels in an RT cell culture (for **f**). Data are presented as the mean ± SEM from three independent experiments. *p < 0.05, **p < 0.01, ***p < 0.001, ****p < 0.0001. (**g**) Comparison of DEGs identified for neonatal, fetal^11^, and juvenile^2^ RT cells. (**h**) Comparison of DEGs identified for neonatal, fetal^11^, and juvenile^2^ SCs. The total numbers of upregulated DEGs in each dataset are presented inside the circles; the numbers of overlapped DEGs are highlighted in bold; and the percentage of the overlap are presented in parentheses. The red type labels the numbers of DEGs that are common to all datasets.

Bulk RNA-seq of three samples of neonatal RT cell cultures and three samples of neonatal SC cultures was performed. A heatmap of Pearson correlation coefficients between samples is shown in Fig. 2a. According to the principal component analysis (PCA), RT cells and SCs were greatly separated in principal component 1 (PC1) (Fig. 2b). Differential expression analysis of the RNA-seq data identified 20,666 unique genes (Supplementary Data).

We selected the genes with a minimum mean expression level of 5 RPKM in at least one of the cell populations and generated a volcano plot (Fig. 2c). DEGs with FDR (false discovery rate) < 0.05 and with an absolute value of log2 fold change (LFC) > 1 were chosen for the further analysis. Among these DEGs, 858 genes were upregulated in RT cells and 835 genes were upregulated in SCs (Supplementary Data). The volcano plot indicated a trend towards greater LFC absolute values of DEGs upregulated in RT cells (Fig. 2c). Indeed, among DEGs with an absolute value of LFC > 4, 144 genes were upregulated in RT cells and only 4 genes were upregulated in SCs (Fig. 2d). The expression of three genes upregulated in RT cells and four genes upregulated in SCs was validated by RT qPCR in independent samples. qPCR data correlated with RNA-seq data (Fig. 2e,f).

Panther-based Gene Ontology (GO) enrichment analysis^16,17^ was performed to characterize RT cells and SCs. Selected GO terms are presented in Table 1, and complete results of the analysis with statistics can be found in Supplementary Data. The term collagen-containing extracellular matrix and terms associated with plasma membrane bounded cell projections were overrepresented in both cell populations. The latter group of terms might indicate cell migration and movements during the establishment of cell cultures.

**Table 1:** Selected terms of GO enrichment analysis of DEGs between neonatal RT cells and neonatal SCs.

RT cell upregulated DEGs	Fold enrich-ment	No. of genes	SC upregulated DEGs	Fold enrich-ment	No. of genes
**GO cellular component complete**					
DNA replication preinitiation complex (GO:0031261)*	14.44	7	Tight junction (GO:0070160)	3.08	17
Kinetochore (GO:0000776)	2.75	17	Endocytic vesicle (GO:0030139)	2.70	21
Heterochromatin (GO:0000792)	3.28	12	Lysosome (GO:0005764)	2.62	52
SMAD protein complex (GO:0071141)	10.73	4	Late endosome (GO:0005770)	2.57	24
Plasma membrane bounded cell projection (GO:0120025)			Plasma membrane bounded cell projection (GO:0120025)		
Microvillus (GO:0005902)	3.54	14	Lamellipodium (GO:0030027)	2.63	17
Filopodium (GO:0030175)	3.52	13	Actin-based cell projection (GO:0098858)	2.50	21
Microspike (GO:0044393)	20.12	3	Mitochondrial envelope (GO:0005740)	1.76	50
Lamellipodium (GO:0030027)	2.71	18	Collagen-containing extracellular matrix (GO:0062023)	2.22	32
Kinesin complex (GO:0005871)	4.38	8			
Apicolateral plasma membrane (GO:0016327)	6.95	7			
Adherens junction (GO:0005912)	2.79	16			
Actin cytoskeleton (GO:0015629)	2.60	49			
Collagen-containing extracellular matrix (GO:0062023)	2.91	43			
**PANTHER GO-slim biological process**					
Glycerol-3-phosphate metabolic process (GO:0006072)	13.41	3	Lipid metabolic process (GO:0006629)	2.07	34
Double-strand break repair via break-induced replication (GO:0000727)	14.63	6	Glutathione metabolic process (GO:0006749)	6.12	8
DNA replication initiation (GO:0006270)	10.43	7	Response to endogenous stimulus (GO:0009719)	3.15	28
Mitotic cell cycle (GO:0000278)	3.09	31	Cellular response to hormone stimulus (GO:0032870)	4.42	13
Transforming growth factor beta receptor signaling pathway (GO:0007179)	5.55	6	Regulation of plasma membrane bounded cell projection organization (GO:0120035)	3.48	11
BMP signaling pathway (GO:0030509)	4.95	12			
Wnt signaling pathway (GO:0016055)	4.07	17			
Ras protein signal transduction (GO:0007265)	2.76	11			
Embryo development (GO:0009790)	3.45	9			
Proteoglycan metabolic process (GO:0006029)	5.87	7			
Establishment or maintenance of cell polarity (GO:0007163)	4.27	7			
Actin cytoskeleton organization (GO:0030036)	2.59	28			
Plasma membrane bounded cell projection morphogenesis (GO:0120039)	2.90	19			

*FDR < 0.05 for all GO terms.

DEGs upregulated in SCs exhibited an enrichment in categories associated with tight junctions, organelles from the endocytic pathway, mitochondria as well as with lipid metabolism and response to endogenous stimuli including hormones (Table 1). In contrast, DEGs upregulated in RT cells were enriched in terms associated with proliferation, heterochromatin, actin cytoskeleton, adherens junctions and cell polarity. They also showed an overrepresentation of genes involved in TGF beta, BMP, and Wnt signaling pathways and genes important for embryonic development (Table 1).

As we especially focused on the specification of RT cells, we used GO and Panther databases to identify DEGs with transcription factor (TF) activity (Table 2). Besides *Pax8*, RT cells exhibited increased expression of *Sox17* described in the RT recently^2^. *Emx2* and *Pbx1* participating in embryonic gonad development^18,19^ as well as *Tbx1*, *Tbx2*, and *Tead2* contributing to formation of various organs^20,21^ were all increased in an RT cell culture. Genes for three members of ID family of TFs were also upregulated in RT cells. It was previously reported that the upregulation of ID TFs dramatically increased SC proliferative activity^22^.

**Table 2:** Selected DEGs between neonatal RT cells and neonatal SCs classified according to GO molecular function complete database and Panther Protein Class database.

Term	Cell type	Gene names
**DNA-binding transcription factor activity (GO:0003700), transcription factor (PC00218)**	RT cells	**68:** *Aebp1, ****Id3****, ****Pax8****, Etv4, Litaf, ****Tbx2****, Jun, ****Id1****, ****Tead2****, Nfix, Peg3, Smad3, ****Emx2****, ****Tbx1****, Arnt2, Patz1, Nfib, Cebpb, ****Pbx1****, ****Sox17****, E2f8, Atoh8, Smad6, Zfp422, Zfp57, Plscr2, ****Id4****, Smad5, Nr2f2, Zfp623, Plagl1, Hoxb7, Mybl2, Sall2, Mycn, Meis2, Sox11, Tshz1, Mxd3, Tcf7l1, Meis1, Pbx3, Nr2f1, Maf, Zbtb8a, Zfp467, Tfcp2l1, Irf5, Tead3, Pou6f1, Zfp101, Zfp651, Hmga2, Hoxb8, Rarb, Hey2, Twist1, Tcf4, Hoxd8, Gm14403, Lef1, Glis3, Zfp618, Hoxb6, Zbtb8b, Zfp9, Nr0b2, Hoxc6*
	SCs	**45:** *Creb3, ****Dmrt1****, ****Sox9****, ****Stat1****, Gprasp2, Cebpd, Stat3, Stat6, ****Thra****, Zmym3, Elf2, Klf4, Gprasp1, Jade3, Junb, Nr1d1, Ddit3, Nfkb2, Brpf3, Csrnp1, Mef2c, Tgif1, Osr1, Jade1, Arid3c, Atf6, Zfp239, Elf1, Fosl1, Zbtb43, Nr4a1, Foxq1, Foxs1, Egr1, Klf9, Atf3, Relb, Klf5, Dbp, Mxd1, Meis3, Npas4, Sox6, Fos, Crebl2*
**receptor ligand activity (GO:0048018), intercellular signal molecule (PC00207)**	RT cells	**40: *Gdf15****, ****Tgfb2****, Ptn, Gas6, S100a4, Stc2, Tmpo, ****Tgfb3****, Nbl1, ****Fgf18****, Hmgb2, Rcan2, Mdk, ****Bmp4****, ****Bmp6****, Fbn2, Sfrp2, ****Fgf9****, Sema3f, Adm, Tnfsf13, Hbegf, Osgin1, Wnt5a, Wnt4, Gdf6, Cxcl16, Cdc42ep2, Il34, Cx3cl1, Ccl2, Il16, Nrg1, Il1rn, Efna4, Egfl7, Slit2, Wnt5b, Gdf11, Dlk1*
	SCs	**17:** *Timp1, Sema6c, Lgals3, Edn1, Pdzd2, Sema5a, Tnfsf12, Sema3d, ****Fgf13****, Lif, Crlf2, ****Inhba****, Osgin2, ****Bmp2****, ****Tgfb1****, ****Inha****, Wnt6*

DEGs are listed in order of decreasing mean RPKM value.

DEGs mentioned in the text are highlighted in bold.

SCs exhibited increased expression of *Dmrt1*, *Sox9*, *Stat1,* and *Thra* (Table 2). *Dmrt1* and *Sox9* were shown to be crucial for maintenance of SC fate^23,24^, and *Stat1* and *Thra* were reported to be important regulators of SC function^25,26^.

Considering the importance of intercellular communications between RT cells and SCs^1,2^, we also identified DEGs demonstrating receptor ligand activity (Table 2). Different members of TGFb, FGF and BMP families of signaling molecules were upregulated in both cell populations. RT cells showed increased expression of *Gdf15* previously identified in adult RT cells^12^. Genes encoding activin and inhibin subunits were upregulated in SCs.

Manual search of SC upregulated DEGs revealed additional SC specific genes, such as *Fshr*^27^, *Dhh*^28^, *Ctsl*^29^, *Shbg*^30^, *Clu*^31^, *Gstm6*^32^, and *Cst12*^33^.

### Comparative analysis of DEGs between RT cells and SCs of various ages

To examine transcriptomes of RT cells and SCs of different ages we used publicly available data obtained in other studies. First, we analyzed scRNA-seq data on juvenile (14 dpp) wild-type mice from the study by Uchida et al.^2^. We generated Uniform Manifold Approximation and Projection (UMAP) plot with 18 cell clusters (Supplementary Fig. S3) and identified clusters of RT cells and SCs using the same criteria as in the original study^2^. Specifically, clusters 2, 3, 11, and 14 highly expressed *Sox9*. Cells from clusters 2, 3, and 11 were defined as SCs. Cluster 14 which also exhibited increased expression levels of *Pax8*, *Cdh1* and *Krt8*, was defined as an RT cell cluster (Supplementary Fig. S3). Further analysis identified 1791 genes differentially expressed between RT cells and SCs with Padj value < 0.05 (Supplementary Data).

We also used scRNA-seq data on fetal mice from the study by Mayère et al.^11^. In this case, DEGs between fetal RT cells (XY late supporting-like cells) and fetal SCs had already been identified by the authors^11^.

Next, we performed pairwise comparisons of DEG lists obtained for fetal^11^, neonatal (the current study), and juvenile cells^2^. The results are presented as Venn diagrams (Fig. 2g,h). The lists of overlapped DEGs can be found in Supplementary Data. As it is shown in Fig. 2g,h, 86 DEGs upregulated in RT cells and 79 DEGs upregulated in SCs are common for all three datasets, which means that these DEGs are permanently differentially expressed between RT cells and SCs throughout testis development and in culture.

As we suggest that cell lineage markers and key genes for the specification of both RT cells and SCs may be among these DEGs, we examined the DEG products using the Panther Protein Class database (Table 3). A non-coding RNA *Meg3* from the list of RT upregulated DEGs was excluded from the analysis. The same was done to *Gm14226* from the SC list as it encoded a viral or transposable element protein. As many as 18 DEGs out of 85 DEGs in the RT list encoded TFs such as PAX8, EMX2, PBX1, ID, and TEAD2 (Table 3). However, only *Dmrt1* and *Mef2c* encoded TFs in the SC list. *Mef2c* was not previously described in SCs whereas *Dmrt1* was identified as a crucial factor in SC fate maintenance, as it was mentioned above. Our immunofluorescence data confirmed the higher level of DMRT1 expression in SCs compared with that in RT cells (Fig. 1e, inset). Genes of transcription cofactors *Cited1* and *Cited2* were also permanently upregulated in SCs with *Cited2* known to be involved in embryonic testis development^34^.

**Table 3:** Classification of DEGs permanently upregulated in RT cells and SCs according to the Panther Protein Class database.

RT cells (85 DEGs)	SCs (78 DEGs)
**Transcription factor (TF, PC00218) and cofactor (PC00217)**	
**18 TFs:** homeodomain TFs *Pax8*, ***Emx2*** ^1^, *Pbx1* ^2^, *Meis2*, ***Meis1***; zinc finger TFs ***Peg3***, *Nr2f2*, ***Tshz1***, ***Nr2f1***; bHLH TF *Tcf4* ^3^; basic leucine zipper TFs *Jun* ^4^, *Maf*; other TFs *Id3* ^5^, ***Id1*** ^5^, *Tead2*, *Nfib*, *Id4* ^5^, *Smad5* ^6^ **3 cofactors:** Zfp503*, Greb1* ^7^, Ssbp2*	**2 TFs:** *Dmrt1* ^8^, ***Mef2c*** **3 cofactors:** *Cited2* ^9^, ***Cited1*** ^10^, Basp1* ^10^
**Chromatin/chromatin-binding, or -regulatory protein (PC00077)**	
**1:** *Cbx6*	**3: *Sin3b*** ^10,11^, histone demethylases ***Phf8***, ***Kdm7a***
**Intercellular signal molecule (PC00207)**	
**7: *Tgfb2*** ^12^, *Ptn*, *Tgfb3*, BMP antagonist Nbl1*; *Mdk*, *Bmp4*, Rarres2*	**4: *Sema6c***, Dhh* ^13^, *Inha* ^10,14^, *Wnt6* ^15^
**Transmembrane signal receptor (PC00197)**	
**6: *Fgfr2*** ^16^, *Il6st*, *Kdr* ^17^, coreceptor Nrp1*, G-protein coupled receptor *Gprc5c*; ***Epha4***	**2:** *Axl* ^18^, ***Plxnc1*** ^19^
**Cytoskeletal protein (PC00085)**	
**7:** *Myl9*, *Crip2*, *Ezr* ^20^, ***Myh10***, Palm*, Nav2*, *Pdlim2*	**5:** *Arpc1b* ^21^, *Tubb3* ^10,22^, *Dynlt1a* ^23^, *Myo7a* ^24^, Wipf3* ^25^
**Protein-binding activity modulator (PC00095)**	
**9:** protease inhibitors ***Timp2*** ^26^, ***Fstl1***, *Igfbp4*, *Serpinf1*, ***Igfbp5***; *Ccnd1* ^27^, Rho GTPase-activating proteins *Arhgap42*, *Dlc1*; Slit3*	**5:** small GTPases ***Rab31***, ***Rnd2***, cysteine proteinase inhibitor Cst12* ^10,28^; Cdc42se1*, heterotrimeric G-protein ***Gng12***
**Scaffold/adaptor protein (PC00226)**	
**8:** *Tspan3*, ***Sorbs2*** ^29^, ***Mical2***, *Spry1*, *Cdc42ep3*, *Mpdz*, *Tspan13*, ***Sh3kbp1***	**2:** *Eps8* ^30^, ***Tulp2*** ^31^
**Metabolite interconversion enzyme (PC00262)**	
**4:** dehydrogenase *Aldh1a3*, transaminase *Bcat1*, phosphodiesterase *Gdpd2*, catalase Cygb*	**15:** oxidoreductases *Me1*, ***Akr1b8***, *Akr1b3* ^10^, *Akr1cl* ^32^, *Cox7a1*; transferases ***Gstm6*** ^10,33^, ***Gatm*** ^10^, ***Gstm7*** ^10^, ***Bcat2***, *Nmt2*, ***Etnk2*** ^10,34^, *St3gal4*; decarboxylase ***Uxs1***, hydrolase Tmem86a*, ligase *Acsbg1*
**Protein modifying enzyme (PC00260)**	
**4:** protease *Aebp1*, phosphatases ***Dusp5*** and *Ptpn13*; ubiquitin-protein ligase ***Zmiz1***	**8:** cysteine protease *Ctsl* ^10,35^, ubiquitin-protein ligases ***Rnf181***, ***Trim47***, ***Dtna***, *Fbxo17*, ***Colgalt1***, ***Zdhhc2***; kinase *Pak3* ^36^
**Transporter (PC00227)**	
**5:** Tmem176b*, Tmem176a*, *Slc48a1*, ***Fxyd1***, *Atp8a1*	**6:** *Slc45a4*, ***Tmem184a*** ^10,37^, ***Ttyh1*** ^10,38^, *Slc4a2* ^39^, ***Fxyd6*** ^10^, *Slco3a1* ^40^
**Membrane traffic protein (PC00150)**	
**2: *Sorl1***, Golgi protein Scoc*	**3:** *Vapa* ^41^, ***Cltb*** ^42^, ***Reep1*** ^43^
**Transfer/carrier protein (PC00219)**	
**1: *Apoe*** ^44^	1: Shbg* ^45^
**Defense/immunity protein (PC00090)**	
**2:** *Ifitm2*, *Pltp*	**3:** *Lsr*, ***Defb19*** ^10,46^, *Defb36* ^10,43^
**cell adhesion molecule (PC00069) 3:** *Bcam*, ***Podxl***, ***Ncam1***	chaperone (PC00072) 2: Clu* ^10,47^, ***Hspb1*** ^48^
**extracellular matrix protein (PC00102)** **1:** *Fbn2*	**calcium-binding protein (PC00060)** **3: *Efhd2*** ^10^, Stim1* ^49,50^, Unc13b*
**RNA metabolism protein (PC00031)** **1: *Rbms3***	**mitochondrion function** **3:** Chchd10* ^10^, Chchd2* ^10^, Nudt19*
**Unclear function (unclassified)**	
**3: *Ctxn1***, *Eva1b*, ***Nrep***	**8: *Bex4***^51^, ***Bex2*** ^10^, *Wsb2*^52^, ***Aard*** ^10,53^, ***AU015836*** ^10^, ***Syap1***, ***Mro*** ^10,54,55^, ***Trank1***^56^

*Manually classified DEGs. The list of references is in Supplementary Data. The references cite studies that reported expression of a gene in the SC lineage or in seminiferous tubules/testis cords. Bold type labels DEGs in the RT list that could be downregulated by DMRT1 and DEGs in the SC list that could be upregulated by DMRT1 (according to^37,38^). DEGs are listed in order of decreasing mean RPKM value.

As opposed to the situation with TFs, 23 out of 78 DEGs in the SC list encoded enzymes (PC00262 and PC00260) whereas only 8 DEGs in the RT list were enzyme genes (Table 3). SC enzymes included GSTM6 and GSTM7, CTSL, and 6 ubiquitin-protein ligases, whereas ALDH1A3, which was responsible for the synthesis of all-trans retinoic acid^35^, was present in the RT list.

A considerable number of DEGs permanently upregulated in RT cells encoded proteins involved in cell signaling such as growth factors TGFB2, 3 and BMP4 and transmembrane signal receptors FGFR2 and KDR. Genes encoding intercellular signal molecules SEMA6C, DHH and INHA and a transmembrane receptor AXL were permanently upregulated in SCs (Table 3, PC00207 and PC00197). In both DEG lists there were genes associated with intracellular signaling through small GTPases with *Cdc42ep3*, *Arhgap42*, *Dlc1*, and *Slit3* upregulated in RT cells and *Rab31*, *Rnd2*, and *Cdc42se1* upregulated in SCs (Table 3, PC00095, PC00226). Genes encoding specific microtubule-associated proteins TUBB3 and DYNLT1A (PC00085), calcium-binding proteins, chaperones (CLU, HSPB1), and proteins related to mitochondrion function were present in the SC list. Genes encoding proteins related to extracellular matrix and cell adhesion, such as BCAM, PODXL, and 5 protease inhibitors (PC00095), were present in the RT list (Table 3).

A manual search of published articles showed that 50 DEGs from the SC list and 17 DEGs from the RT list were previously described in SC lineage (Table 3, references for Table 3 can be found in Supplementary References), however the functional significance of many of them in the testis was not defined. The SC list from the current study also strongly corresponded to the gene list from the study utilizing the RiboTag approach to identify SC genes that were actively translated^36^.

Considering that a crucial TF DMRT1 was one of the only two TFs in the SC list, we conducted an additional comparative analysis using two recent studies on the mechanisms of DMRT1 action. One of them performed bulk RNA-seq to examine the changes in the transcriptome of cultured granulosa cells after DMRT1 activation^37^. The other study utilized the same method for identifying the differences in gene expression between neonatal SCs isolated from mice homozygous and heterozygous for a *Dmrt1* null mutation^38^. We used the lists of DEGs that could be upregulated or downregulated by DMRT1 according to these studies, combined them, excluded contradictory genes that exhibited opposed dynamics, and compared with the lists of RT and SC permanently upregulated genes (Supplementary Data). It was found that DMRT1 could downregulate 31 DEGs from the RT list and upregulate 45 DEGs from the SC list including transcriptional regulators *MEf2c*, *Cited1*, and *Basp1* (Table 3, DMRT1 candidate targets were highlighted in bold).

### Expression of RT and SC genes in adult testicular cultures

**Figure 3:**
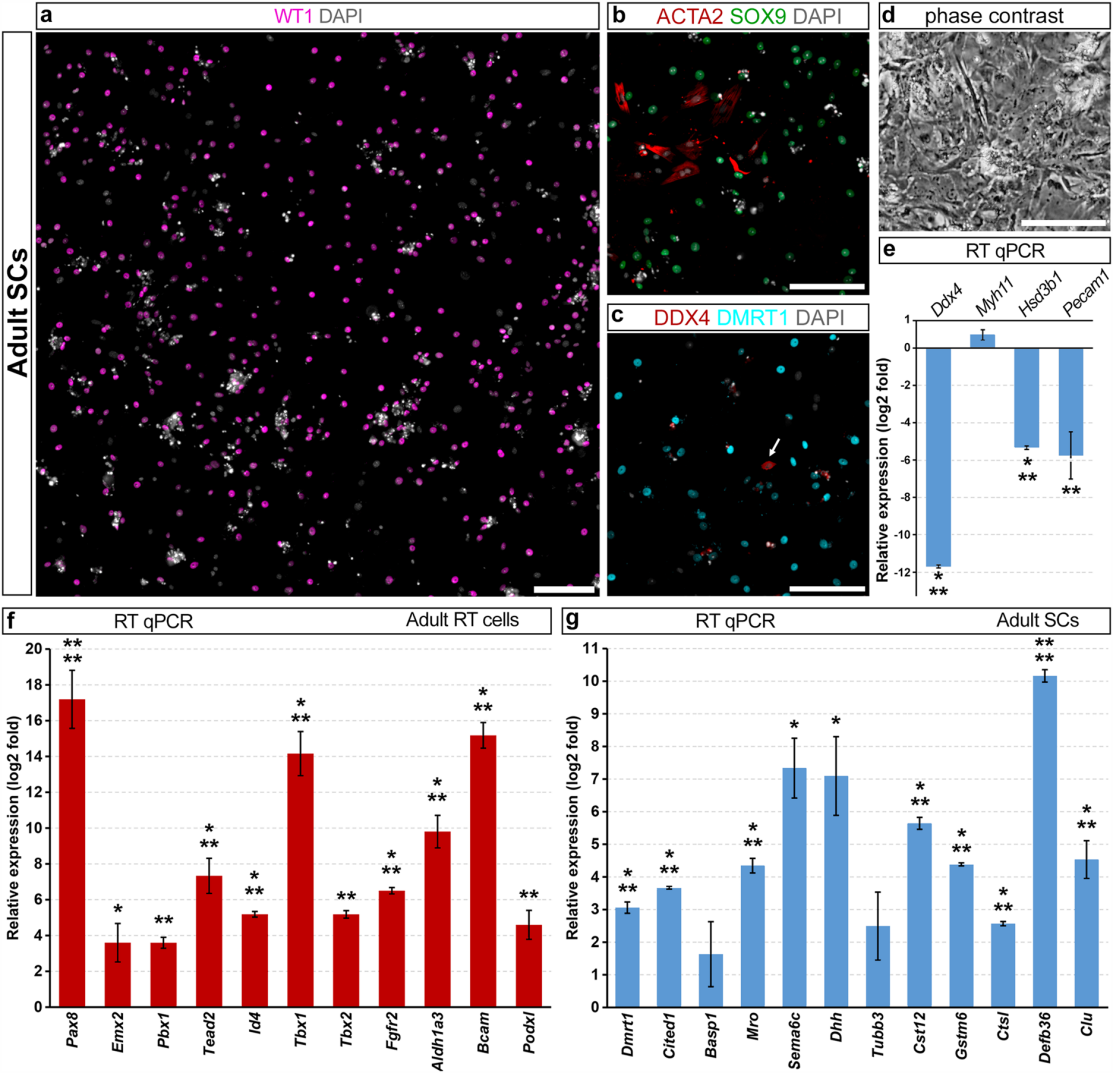
Characterization of an adult SC culture and confirmation of the RT and SC gene lists using adult cell cultures. (**a**–**c**) Immunofluorescent staining of an adult SC culture on day 8 for WT1 (**a**), for ACTA2 and SOX9 (**b**), and for DDX4 and DMRT1 (**c**). Images were stitched from several adjacent fields of view. An arrow in (**c**) points to a germ cell. (**d**) Morphological appearance of an adult SC culture. (**e**) mRNA levels of markers of different testicular cell populations in an adult SC culture relative to that in adult testicular tissue (the zero line). (**f**) mRNA levels of genes from the RT list in an adult RT cell culture relative to that in an adult SC culture (the zero line). (**g**) mRNA levels of genes from the SC list in an adult SC culture relative to that in an adult RT cell culture (the zero line). Data in (**e–g**) are presented as the mean ± SEM from three independent experiments. *p < 0.05, **p < 0.01, ***p < 0.001, ****p < 0.0001. Scale bars: 100 μm.

To examine if the lists of RT and SC permanently upregulated genes were appropriate for the adult cells, we compared mRNA levels of selected genes in cultures of adult RT cells and SCs using RT qPCR. Establishment of a pure culture of adult RT cells was described above (Fig. 1h–l). For an adult SC culture, we used a method developed in the study by Saewu et al*.*^39^ which was based on serial enzymatic digestions. In our hands, the SC number, identified by WT1, SOX9 and DMRT1 staining, was 64.8 ± 6.4% on day 8 (Fig. 3a), and the main admixture was peritubular myoid cells (Fig. 3b). Germ cells were present in the culture at first, but they were gradually removed during medium replacements. So, only a few germ cells, identified by DDX4 staining (Fig. 3c, arrow), and a small amount of germ cell debris (Fig. 3a,d) remained on day 8. According to RT qPCR, markers of germ, Leydig, and endothelial cells were expressed at lower levels compared with those in testicular tissue, whereas a myoid cell marker *Myh11* was slightly upregulated (Fig. 3e).

Despite the fact that the culture lengths of neonatal and adult cells were different, all of the selected genes from the RT list were strongly upregulated in an adult RT culture compared with an adult SC culture (Fig. 3f). We also measured the expressions of TF genes *Tbx1* and *Tbx2*. They were not included in the RT list, but *Tbx1* was upregulated in juvenile RT cells^2^, *Tbx2* was elevated in fetal RT cells^11^, and both of them were increased in neonatal and adult RT cells. So, we suggested that they might be important regulators of RT function. Selected genes from the SC list were upregulated in an adult SC culture compared with an adult culture of RT cells, although for two of them, *Basp1* and *Tubb3*, the increase was statistically insignificant (Fig. 3g). Similarly to RNA-seq data, the genes from the RT list exhibited higher expression fold changes than the genes from the SC list.

These results suggest that at least some of the genes from the lists of RT and SC permanently upregulated genes are valid for adult cells and also for prolonged culture.

## Discussion

In our previous report^12^, we used RT cell cultures that also contained peritubular myoid cells and SCs, with the numbers of these cells being especially high in neonatal RT cell cultures. That hindered the interpretation of the experiments and the culture propagation. In the current study, we developed a procedure for establishing a pure culture of RT cells. Specifically, we used FACS to sort CDH1^+^ cell fraction from a testicular cell suspension. Applying this approach, we verified our previous results, according to which a substantial number of RT cells express TF DMRT1, which was earlier observed only in SCs and germ cells^40^.

Next, we employed pure cultures of neonatal RT cells and SCs^13^ to compare their gene expression by bulk RNA-seq. A general view of RNA-seq data (Fig. 2c,d) showed that a considerable number of DEGs upregulated in RT cells exhibited great values of expression fold change, which suggested that these genes were expressed in SCs at extremely low levels. As opposed to that, fold change values tended to be lower for DEGs upregulated in SCs. On the one hand, this could reflect the presence of at least two subpopulations of RT cells, one of which is more similar to SCs. On the other hand, this could indicate that RT cells express many SC genes at some basic level, while the phenotype and function of RT cells are mainly determined by specific RT genes.

**Figure 4:**
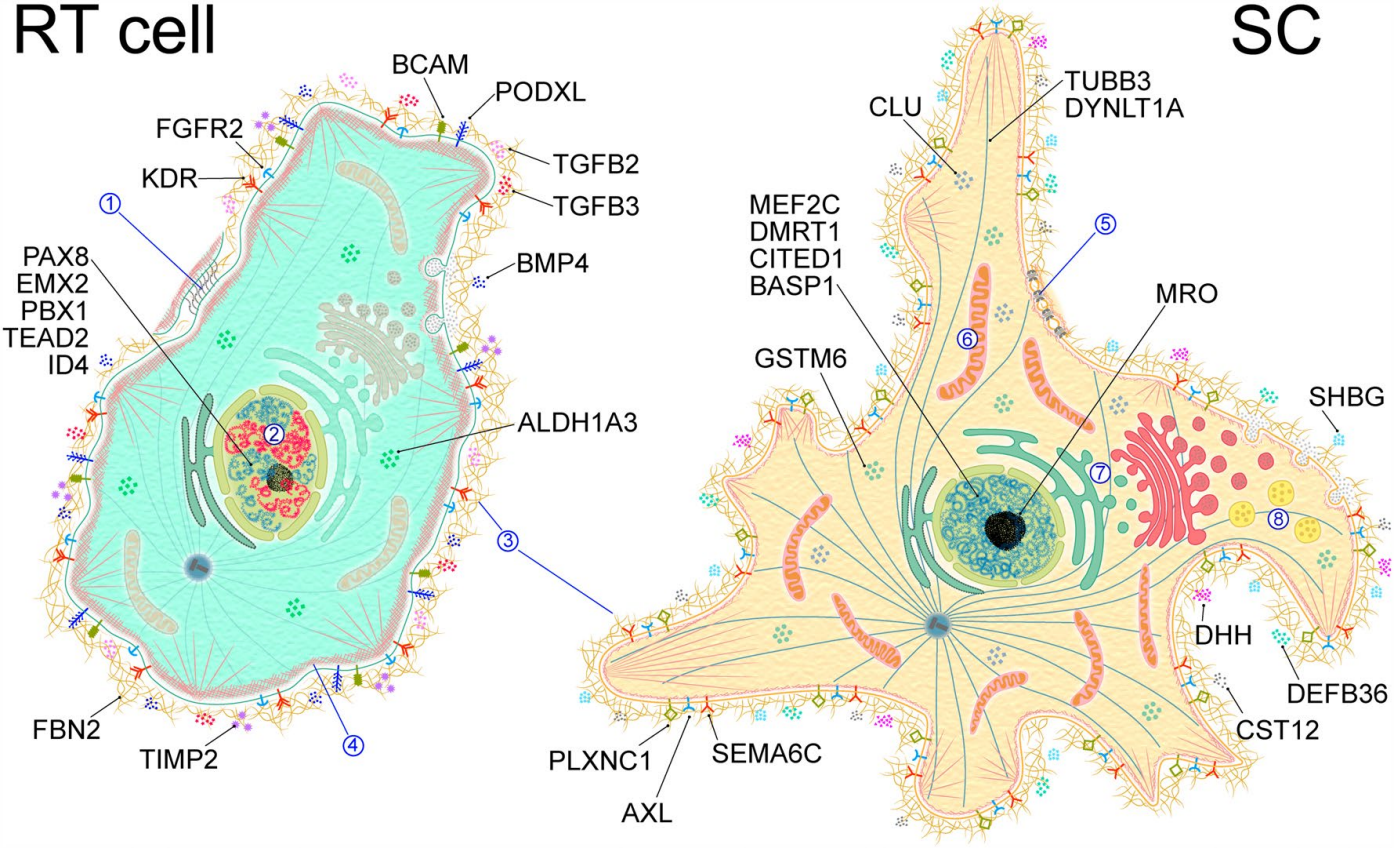
A schematic picture illustrating the differences between RT cells and SCs. Numbers in circles refer to selected terms of the functional analysis of DEGs between neonatal RT cells and SCs. 1—adherens junction, 2—DNA replication, 3—extracellular matrix, 4—actin cytoskeleton, 5—tight junction, 6—mitochondrion, 7—lipid metabolism, 8—endocytic pathway. Proteins encoded by some permanent DEGs from Table 3 are shown.

Functional enrichment analysis of the DEGs showed that genes upregulated in neonatal SCs indicated enrichment in GO terms related to tight junctions, lipid and glutathione metabolism, and mitochondria, which were all consistent with SC functions in the testis^41^. Enrichment in terms associated with the endocytic pathway could point to the transformation of the cell membrane during SC differentiation that occurs postnatally^42^. DEGs upregulated in neonatal RT cells indicated enrichment in terms related to proliferation, cell polarity, and actin cytoskeleton, which characterized RT cells as highly proliferating epithelial cells. RT upregulated DEGs were also strongly enriched in terms of various signaling pathways and in the term embryo development, which could indicate that some morphogenetic processes occur in the RT cell population. Figure 4 shows a graphic interpretation of the results of the functional analysis.

It was shown earlier^12^ that RT cells express WT1, SOX9, DMRT1, and AMH, which are all often used as SC markers. PAX8, CDH1, and KRT8 were reported to be RT cell markers^12,14^, although two of them, CDH1 and KRT8, were demonstrated to gradually disappear from cultured RT cells^12^. scRNA-seq analyses of fetal and juvenile testes revealed some additional genes upregulated in RT cells, such as *Ncam1*, *Tbx2*, and *Ennp2* for fetal cells^11^ and *Sox17* for juvenile cells^2^. Microarray analysis of adult testes identified that various signaling factors, such as *Fgf9*, *Bmp4*, *Notch1*, and *Wnt*, are upregulated in the RT epithelium^1^. However, it was unclear if all these genes were constantly upregulated in the RT during fetal and postnatal development or if they remained in RT cells in culture. So, there was a shortage of reliable universal markers for distinguishing these two cell populations.

A comparative analysis of DEGs between RT cells and SCs of different ages, both in vivo and in vitro, could address the issue. There are studies examining only the SC transcriptome^25,36^, but they are not suitable for comparison to RT cells. There is a study that performed scRNAseq analysis on all testicular cells throughout the perinatal period^43^. However, it was not designed to specifically examine the RT cell population, which is extremely small and needs special efforts to study. As far as we know, there are only two studies^2,11^ examining the transcriptome of mouse RT cells using scRNAseq, which may be suitable for our comparative purposes.

So, in this study, we used scRNAseq datasets from one study examining juvenile testes^2^ and DEGs between RT cells and SCs from another study examining fetal testes^11^, and we compared these data with our list of DEGs between neonatal cultures of RT cells and SCs. As a result, we generated lists of DEGs permanently upregulated in each cell population throughout testis development and in culture (Table 3, Fig. 4). We also showed using RT qPCR that at least some of these DEGs are valid for adult cultures of RT cells and SCs.

We suggest that some of these genes may become new markers for distinguishing between RT cells and SCs. However, caution needs to be taken with SCs from the transition region between seminiferous tubules and the RT (the Sertoli valve), which appears in testes from the juvenile stage onwards. For example, it was reported that *Fgfr2* from the RT list was expressed not only in the RT but also in the Sertoli valve^1^.

Examination of DEGs permanently upregulated in RT cells revealed various TFs (Table 3), which could participate in the specification of the RT cell population. *Dmrt1* was one of the only two TF genes permanently upregulated in SCs. According to the studies on DMRT1 function^37,38^, a substantial number of RT and SC permanently upregulated genes could be regulated by DMRT1 (Table 3). So, DMRT1 may contribute to the differences between these two cell populations.

In summary, in the current study, we performed a comparative analysis of DEGs between RT cells and SCs obtained from mice of various ages. For this purpose, we used our original data and the results of previous studies^2,11^. We generated lists of DEGs permanently upregulated in each cell population throughout testis development and in culture. We suggest that some of these genes could be used as cell population markers. We also hypothesize that these genes participate in RT and SC specification.

## Methods

### Animals

C57Bl/6 J mice were purchased from the “Stolbovaya” breeding center of the Scientific Centre of Biomedical Technologies (Russia) and bred in the institutional animal facility to obtain 5–6 dpp male pups and adult 2–4-month-old male mice. The animals were housed in accordance with the European Convention for the Protection of Vertebrate Animals, and all the experiments were approved by the Animal Care and Use Committee of the Koltzov Institute of Developmental Biology RAS.

### Testicular cell isolation

For neonatal cell isolation testes from 13 to 17 pups were harvested and each testis was cut into a smaller RT region and a larger seminiferous tubule (ST) region, which were further digested separately. Testis fragments were then decapsulated, dissected and digested in 1 ml of collagenase type IV (1 mg/mL, Worthington) and DNase I (0.1 mg/mL, Sigma) in DMEM/F12 medium with penicillin/streptomycin at 37 °C on an orbital shaker (210 rpm) for 30 min. The tubes with testis fragments were accurately shaken to obtain separate tubules, which were then washed in Hanks' Balanced Salt Solution (HBSS) and centrifuged at 30 g for 1 min to remove interstitial cells. Next, the testis fragments were incubated in 1 mL of collagenase, DNase I and hyaluronidase I-S (2 mg/mL; Sigma) twice, for 30 min and 1 h, under the same conditions. The tubules were washed and centrifuged at 30 g for 1 min between the digestions. After the third digestion, the fragments were dissociated into single cells by repeated pipetting, then were passed through a 40 µm cell strainer and centrifuged at 500×*g* for 5 min. The pellets were resuspended in DMEM/F12 medium with 2% fetal bovine serum (FBS), penicillin/streptomycin, sodium pyruvate, and 0.1 mg/ml DNase I (a sorting medium). The yields were 0.9–1.8 × 10^5^ cells/testis for RT regions and 3 × 10^5^ cells/testis for ST regions.

For adult cell isolation testes from 3 mice were divided into RT and ST regions. Adult RT cells were obtained from RT regions using the procedure described above with 2-ml volume of enzyme solutions. The cell yield was 8–11 × 10^5^ cells/testis. Adult SCs were obtained from ST regions according to the method described in the study by Saewu et al.^39^ with 5-ml volume of enzyme solutions and with the yield of 7.5 × 10^6^ cells/testis.

### Cell staining and FACS

Testicular cells from pup ST and RT regions and from adult RT regions were adjusted to a concentration of 10^7^ cells/ml in the sorting medium. Cells from RT regions were incubated with PE-conjugated rat IgG1, kappa antibody against CDH1 (10 μg/mL, Biolegend, # 147304) in the dark for 45 min on an orbital shaker (210 rpm). Cells from pup ST regions were stained with PE-conjugated rat IgG2a, kappa antibody against PDGFRA (6.7 μg/mL, Biolegend, # 135905) under the same conditions. PE-conjugated rat IgG1, kappa antibody (10 μg/mL, Biolegend, # 147304) and PE-conjugated rat IgG2a, kappa antibody (6.7 μg/mL, Biolegend, # 400507) were used as isotype controls respectively. Finally, the stained cells were washed by centrifugation at 500×*g* for 5 min. FACS was performed using a BioRad S3e Cell Sorter. FACS data were analyzed using Cytospec software (http://www.cyto.purdue.edu/Purdue_software).

### Cell cultures

Sorted cells were plated in Matrigel-coated culture plates at a concentration of 1.1 × 10^4^ cells/cm^2^ for CDH1^+^ cell fraction from pup RT regions, 1.6 × 10^4^ cells/cm^2^ for CDH1^+^ fraction from adult RT regions, and 7 × 10^4^ cells/cm^2^ for PDGFRA^-^ fraction from pup ST regions. Cells were cultured in DMEM/F12 medium with 1% FBS, alanyl-glutamine, sodium pyruvate, insulin-transferrin selenium (ITS), penicillin/streptomycin, 10 mM Y-27632 (Abcam), 0.5 mM A-83-01 (Sigma), and 3 mM CHIR99021 (Sigma). Adult SCs were plated in Matrigel-coated plates at a concentration of 2.3 × 10^6^ cells/cm^2^ in DMEM/F12 serum-free medium with alanyl-glutamine, sodium pyruvate, ITS, penicillin/streptomycin, and epidermal growth factor (2.5 ng/mL). Pup cells were cultured for 3 days without medium change. Adult cells were grown for 8 days with the medium changed every other day. All cells were maintained at 37 °C under 5% CO_2_ atmosphere.

### Immunofluorescence and cell counting

For cell staining, pup cells were grown in 96-well culture plates, and 4-well plates were used for adult cells. Culture samples were fixed in 4% paraformaldehyde (PFA) for 10 min, washed in PBS, then blocked and permeabilized with 3% bovine serum albumin (BSA), 0.1% Tween 20 and 0.5% Triton X-100 for 30 min at 37 °C. Primary antibodies against PAX8 (1:300, rabbit, Thermo Fisher Sci, # MA5-32382), SOX9 (1:400, rabbit, Millipore, # AB5535), WT1 (1:200, rabbit, Abcam, # ab89901), and vimentin (1:5000, chicken, Thermo Fisher Sci, # PA1-10003) were applied overnight at 4 °C. Primary antibodies against DMRT1 (1:50, mouse, Santa Cruz, # sc-377167), DDX4 (1:100, rabbit, Abcam, # ab13840) and ACTA2 (1:50, rabbit, Abcam, # ab5694) were applied for 1 h at 37 °C. After washing, samples were incubated with corresponding secondary antibodies conjugated with Alexa Fluor fluorophores (Thermo Fisher Sci, 1:500) for 30 min at 37 °C. Cell nuclei were co-stained with DAPI. Samples were imaged on a Leica Thunder microscope. Samples stained without primary antibodies showed no positive signal. For ACTA2 staining, cells were first stained for a nuclear antigen, imaged and then re-stained for ACTA2 and re-imaged. Subtraction of the nuclear staining from the second set of images was performed in CellProfiler.

The numbers of positively stained cells were counted using CellProfiler with 1.3–5 × 10^3^ cells analyzed for each pup sample and 1.1–1.9 × 10^5^ cells analyzed for each adult sample. Cells from 3 to 5 independent cultures were counted for each experiment. Data were presented as mean ± s.e.m.

For whole-mount staining, RT regions of 6 dpp testes were carefully decapsulated, fixed in 4% PFA for 30 min at 4 °C and processed through a staining procedure as described earlier^44^. Samples were incubated in a mixture of PE-conjugated CDH1 antibody (1:20) and PAX8 primary antibody (1:100) for 72 h at 4 °C. After 24-h washing, samples were incubated with AF 488-conjugated anti-rabbit antibody (1:500, Thermo Fisher Sci, # A-21206) and DRAQ5 (1:500, Thermo Fisher Sci) overnight at 4 °C and then washed. Samples were imaged on a Zeiss LSM 880 confocal microscope. Samples stained with isotype control antibodies showed no positive signal.

### mRNA sequencing and bioinformatics analysis

RNA sequencing of RT and SC culture samples obtained from pup testes in three independent experiments was performed by Evrogen (Moscow, Russia). Cells grown on one well of a 6-well culture plate were used for each sample. Total RNA was extracted using ExtractRNA kit (Evrogen) and was treated with duplex-specific nuclease (Evrogen). The RNA quality was checked by agarose gel electrophoresis. Poly(A) enrichment of the total RNA, cDNA synthesis and preparation of cDNA libraries were performed using TruSeq mRNA Stranded (Illumina). The libraries were sequenced on Illumina NovaSeq 6000 generating from 82 to 105 million 100 bp single-end reads for each sample. FASTQ files were obtained using bcl2fastq v2.20 Conversion Software (Illumina). Reads were trimmed using Trim Galore and Cutadapt^45^ and assessed for quality with FastQC. HiSat2 algorithm^46^ was used to map reads to the GRCm38 genome, and RSeQC^47^ was used to control the alignment quality. Read summarization was performed using FeatureCounts v.2.0.1^48^. The EdgeR R-package v.3.42.4^49^ was used for the data normalization and differential expression analysis as follows: calcNormFactors function (RLE algorithm) was used to normalize read counts, estimateDisp, glmFit, and glmLRT functions were used for identification of DEGs between RT cells and SCs with FDR < 0.05 and |LFC|> 1 significance threshold. RPKM values were also obtained by using EdgeR.

For scRNA-seq data analysis, a data set was mined from NCBI GEO database (https://www.ncbi.nlm.nih.gov/geo/). The GSE190043 dataset contained scRNA-seq data of the 14 dpp cells located at the proximal part of the testis^2^. The data were processed with parameters described in^2^ using Seurat v4.3.0 R package^50^. Clusters 2, 3, and 11 were annotated as wild-type SCs, cluster 14 was annotated as wild-type RT cells. DEGs between SCs and RT cells were filtered out with threshold of Padj < 0.05.

### Functional enrichment analysis and protein classification

Lists of DEGs were analyzed with PANTHER classification system (https://pantherdb.org). Gene Ontology (released 2023-05-10) and PANTHER (version 17.0, released 2022-02-22) databases were used for the enrichment analysis and for classification of DEGs. Overrepresentation Test (released 20230705) tool was selected, and Fisher's exact test with FDR correction was performed. Hierarchy sort was applied to examine and select annotation terms.

### Total RNA isolation and RT qPCR

Cells from one well of a 6-well culture plate were used for RNA isolation. Total RNA was extracted using TRIzol reagent (Thermo Fisher Sci). cDNA was synthesized by a MMLV RT kit (Evrogen, Russia), and RT qPCR was performed in triplicates using SYBR green qPCRmix-HS with ROX (Evrogen) on a StepOnePlus Real-Time PCR System (Applied Biosystems). PCR amplification conditions were as follows: 45 cycles of 95 °C for 15 s, 60 °C for 30 s. Primer sequences (Supplementary Table) were obtained from PrimerBank (https://pga.mgh.harvard.edu/primerbank), and primers were ordered from Evrogen. Hprt was used as a reference gene to calculate ΔCt values. Fold changes of gene expression were calculated using the 2 − ΔΔCt method. Data were presented as mean ± s.e.m of three independent experiments. Statistical significance was determined by a nonparametric Mann–Whitney U test.

### Supplementary Information

Supplementary Information 1.

Supplementary Information 2.

## Data Availability

All relevant data are within the paper and its Supplementary Data files. Bulk RNA-seq data are available through the GEO database with accession number GSE242442.

## References

[1] Imura-Kishi K (2021). Low retinoic acid levels mediate regionalization of the Sertoli valve in the terminal segment of mouse seminiferous tubules. Sci. Rep..

[2] Uchida A (2022). SOX17-positive rete testis epithelium is required for Sertoli valve formation and normal spermiogenesis in the male mouse. Nat. Commun..

[3] Aiyama Y (2015). A niche for GFRα1-positive spermatogonia in the terminal segments of the seminiferous tubules in hamster testes. Stem Cells..

[4] Figueiredo AF, França LR, Hess RA, Costa GM (2016). Sertoli cells are capable of proliferation into adulthood in the transition region between the seminiferous tubules and the rete testis in Wistar rats. Cell Cycle.

[5] Figueiredo AFA (2019). Prepubertal PTU treatment in rat increases Sertoli cell number and sperm production. Reproduction.

[6] Cao Y (2021). Dysregulation of Notch-FGF signaling axis in germ cells results in cystic dilation of the rete testis in mice. J. Cell Commun. Signal..

[7] Combes AN (2009). Three-dimensional visualization of testis cord morphogenesis, a novel tubulogenic mechanism in development. Dev. Dyn..

[8] Nel-Themaat L (2009). Morphometric analysis of testis cord formation in Sox9-EGFP mice. Dev. Dyn..

[9] Omotehara T, Wu X, Kuramasu M, Itoh M (2020). Connection between seminiferous tubules and epididymal duct is originally induced before sex differentiation in a sex-independent manner. Dev. Dyn..

[10] Kulibin AY, Malolina EA (2020). Formation of the rete testis during mouse embryonic development. Dev. Dyn..

[11] Mayère C (2022). Origin, specification and differentiation of a rare supporting-like lineage in the developing mouse gonad. Sci. Adv..

[12] Malolina EA, Kulibin AY (2019). The rete testis harbors Sertoli-like cells capable of expressing DMRT1. Reproduction.

[13] Malolina EA, Galiakberova AA, Dashinimaev EB, Kulibin AY (2022). Establishment of a pure culture of immature Sertoli cells by PDGFRA staining and cell sorting. Mol. Reprod. Dev..

[14] Nagasawa K (2018). Regionally distinct patterns of STAT3 phosphorylation in the seminiferous epithelia of mouse testes. Mol. Reprod. Dev..

[15] Tokuda M, Kadokawa Y, Kurahashi H, Marunouchi T (2007). CDH1 is a specific marker for undifferentiated spermatogonia in mouse testes. Biol. Reprod..

[16] Thomas PD (2022). PANTHER: Making genome-scale phylogenetics accessible to all. Protein Sci..

[17] Gene Ontology Consortium (2023). The Gene Ontology knowledgebase in 2023. Genetics.

[18] Kusaka M (2010). Abnormal epithelial cell polarity and ectopic epidermal growth factor receptor (EGFR) expression induced in Emx2 KO embryonic gonads. Endocrinology.

[19] Schnabel CA, Selleri L, Cleary ML (2003). Pbx1 is essential for adrenal development and urogenital differentiation. Genesis.

[20] Kispert A (2017). T-Box genes in the kidney and urinary tract. Curr. Top. Dev. Biol..

[21] Landin-Malt A, Benhaddou A, Zider A, Flagiello D (2016). An evolutionary, structural and functional overview of the mammalian TEAD1 and TEAD2 transcription factors. Gene..

[22] Chaudhary J, Sadler-Riggleman I, Ague JM, Skinner MK (2005). The helix-loop-helix inhibitor of differentiation (ID) proteins induce post-mitotic terminally differentiated Sertoli cells to re-enter the cell cycle and proliferate. Biol. Reprod..

[23] Matson CK (2011). DMRT1 prevents female reprogramming in the postnatal mammalian testis. Nature.

[24] Barrionuevo FJ (2016). Sox9 and Sox8 protect the adult testis from male-to-female genetic reprogramming and complete degeneration. Elife.

[25] Zimmermann C (2015). Research resource: The dynamic transcriptional profile of Sertoli cells during the progression of spermatogenesis. Mol. Endocrinol..

[26] Holsberger DR, Kiesewetter SE, Cooke PS (2005). Regulation of neonatal Sertoli cell development by thyroid hormone receptor alpha1. Biol. Reprod..

[27] Heckert LL, Griswold MD (1991). Expression of follicle-stimulating hormone receptor mRNA in rat testes and Sertoli cells. Mol. Endocrinol..

[28] Bitgood MJ, Shen L, McMahon AP (1996). Sertoli cell signaling by Desert hedgehog regulates the male germline. Curr. Biol..

[29] Erickson-Lawrence M, Zabludoff SD, Wright WW (1991). Cyclic protein-2, a secretory product of rat Sertoli cells, is the proenzyme form of cathepsin L. Mol. Endocrinol..

[30] Hagenäs L (1975). Sertoli cell origin of testicular androgen-binding protein (ABP). Mol. Cell. Endocrinol..

[31] Bailey R, Griswold MD (1999). Clusterin in the male reproductive system: Localization and possible function. Mol. Cell. Endocrinol..

[32] Beverdam A (2009). Sox9-dependent expression of Gstm6 in Sertoli cells during testis development in mice. Reproduction.

[33] Li Y (2005). Immunolocalization and regulation of cystatin 12 in mouse testis and epididymis. Biol. Reprod..

[34] Buaas FW, Val P, Swain A (2009). The transcription co-factor CITED2 functions during sex determination and early gonad development. Hum. Mol. Genet..

[35] Beedle MT (2019). Sources of all-trans retinal oxidation independent of the aldehyde dehydrogenase 1A isozymes exist in the postnatal testis. Biol. Reprod..

[36] De Gendt K, Verhoeven G, Amieux PS, Wilkinson MF (2014). Genome-wide identification of AR-regulated genes translated in Sertoli cells in vivo using the RiboTag approach. Mol. Endocrinol..

[37] Lindeman RE (2021). The conserved sex regulator DMRT1 recruits SOX9 in sexual cell fate reprogramming. Nucleic Acids Res..

[38] Murphy MW, Gearhart MD, Wheeler A, Bardwell VJ, Zarkower D (2022). Genomics of sexual cell fate transdifferentiation in the mouse gonad. G3.

[39] Saewu A (2020). Primary Sertoli cell cultures from adult mice have different properties compared with those derived from 20-day-old animals. Endocrinology.

[40] Zarkower D, Murphy MW (2022). DMRT1: An ancient sexual regulator required for human gonadogenesis. Sex. Dev..

[41] França LR, Hess RA, Dufour JM, Hofmann MC, Griswold MD (2016). The Sertoli cell: One hundred fifty years of beauty and plasticity. Andrology.

[42] Griswold MD, McLean D, Neill JD (2006). The Sertoli cell. Knobil and Neill's Physiology of Reproduction.

[43] Tan K, Song HW, Wilkinson MF (2020). Single-cell RNAseq analysis of testicular germ and somatic cell development during the perinatal period. Development.

[44] Gassei K, Valli H, Orwig KE (2014). Whole-mount immunohistochemistry to study spermatogonial stem cells and spermatogenic lineage development in mice, monkeys, and humans. Methods Mol. Biol..

[45] Martin M (2011). Cutadapt removes adapter sequences from high-throughput sequencing reads. EMBnet J..

[46] Kim D, Paggi JM, Park C, Bennett C, Salzberg SL (2019). Graph-based genome alignment and genotyping with HISAT2 and HISAT-genotype. Nat. Biotechnol..

[47] Wang L, Wang S, Li W (2012). RSeQC: Quality control of RNA-seq experiments. Bioinformatics..

[48] Liao Y, Smyth GK, Shi W (2014). featureCounts: An efficient general purpose program for assigning sequence reads to genomic features. Bioinformatics.

[49] Robinson MD, McCarthy DJ, Smyth GK (2010). edgeR: A Bioconductor package for differential expression analysis of digital gene expression data. Bioinformatics.

[50] Hao Y (2021). Integrated analysis of multimodal single-cell data. Cell..

