# Supplementary material for: A comparative analysis of genes differentially expressed between rete testis cells and Sertoli cells of the mouse testis
Source: Sci Rep. 2023 Nov 28;13:20896. doi: 10.1038/s41598-023-48149-7 (PMC10684643; doi:10.1038/s41598-023-48149-7)
Supplement: Supplementary file 2 — Supplementary Information 2. [file 41598_2023_48149_MOESM2_ESM.pdf]

Supplementary Information

COMPARATIVE ANALYSIS OF GENES DIFFERENTIALLY EXPRESSED BETWEEN RETE TESTIS CELLS AND SERTOLI CELLS OF THE MOUSE TESTIS

Ekaterina A. Malolina[1]*, Adelya A. Galiakberova[2], Valery V. Mun[1], Marat S. Sabirov[1], Erdem B. Dashinimaev[2, 3], Andrey Yu. Kulibin[1]

[1]Koltzov Institute of Developmental Biology, Russian Academy of Sciences, 119334 Moscow, Russia
[2]Center for Precision Genome Editing and Genetic Technologies for Biomedicine, Pirogov Russian National Research Medical University, Moscow 117997, Russia
[3]Moscow Institute of Physics and Technology (State University), Institutskiy per., 141701, Dolgoprudny, Russia

*Contact e-mail: kate.ma85@gmail.com

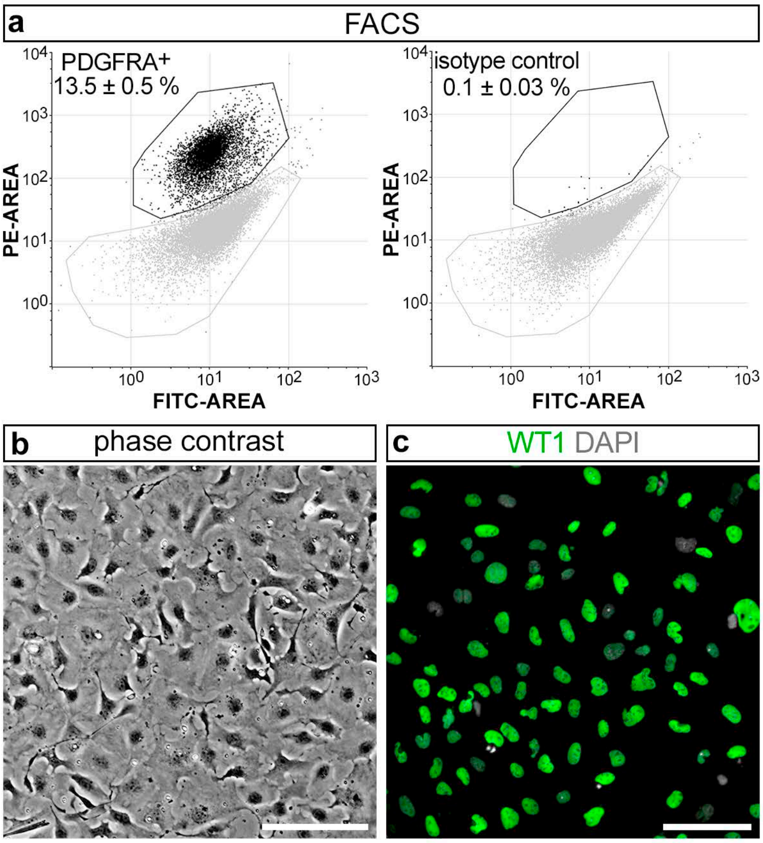

Supplementary Figure S1. Characterization of 5-6 dpp SC culture. (**a**) FACS of cells isolated from seminiferous tubule regions of neonatal testes. Cells were stained with PDGFRA and isotype control antibodies which were labeled with PE. Debris and doublets were excluded from the analysis. The percentage of PDGFRA+ cells was presented as the mean ± SEM from three independent experiments. (**b**) Morphological appearance of a neonatal SC culture on day 3. (**c**) Immunofluorescent staining of the culture for WT1. Scale bars: 100 µm.

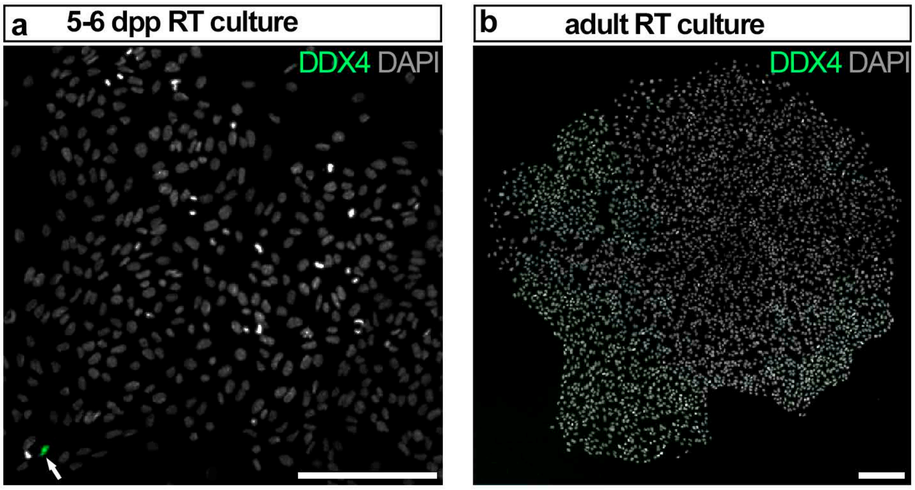

Supplementary Figure S2. Immunofluorescent staining of RT cell cultures for DDX4. An arrow points to a DDX4+ cell. Scale bars: 100 µm.

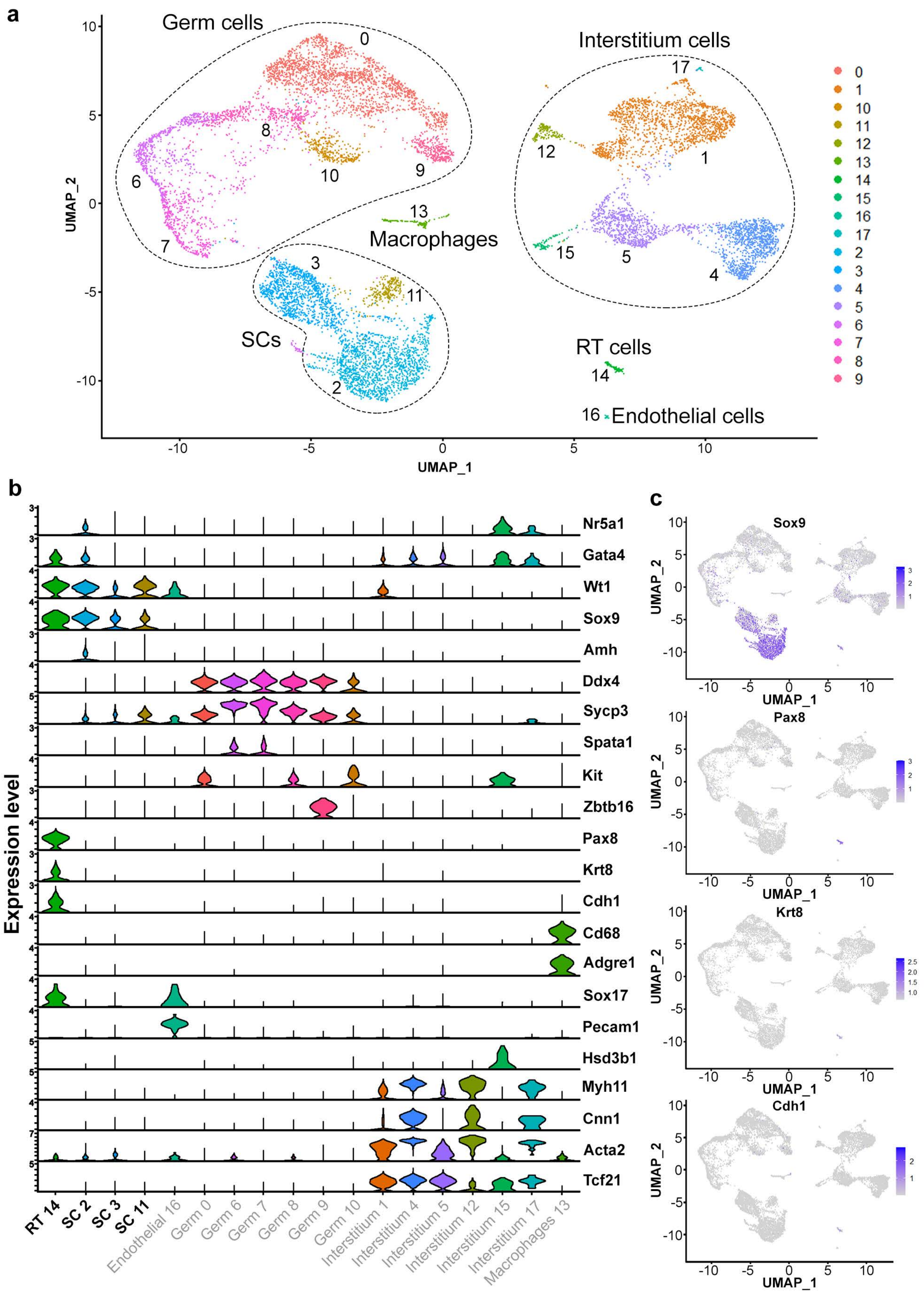

Supplementary Figure S3. scRNA-seq analysis of juvenile (14 dpp) wild-type testes (raw data obtained from GSE190043 dataset). (**a**) A UMAP plot with 18 cell type clusters. (**b**) Violin plots showing the expression levels of marker genes for each cell type. (**c**) UMAP plots showing the distribution of marker genes for RT cells and SCs.

SUPPLEMENTARY REFERENCES FOR TABLE 3

1. Kusaka, M. *et al.* Abnormal epithelial cell polarity and ectopic epidermal growth factor receptor (EGFR) expression induced in Emx2 KO embryonic gonads. *Endocrinology.* **151,** 5893-5904 (2010).
2. Schnabel, C. A., Selleri, L. & Cleary, M. L. Pbx1 is essential for adrenal development and urogenital differentiation. *Genesis.* **37,** 123-130 (2003).
3. Muir, T., Sadler-Riggleman, I., Stevens, J. D. & Skinner, M. K. Role of the basic helix-loop-helix protein ITF2 in the hormonal regulation of Sertoli cell differentiation. *Mol. Reprod. Dev.* **73,** 491-500 (2006).
4. Hamil, K. G., Conti, M., Shimasaki, S. & Hall, S. H. Follicle-stimulating hormone regulation of AP-1: inhibition of c-jun and stimulation of jun-B gene transcription in the rat Sertoli cell. *Mol. Cell. Endocrinol.* **99,** 269-277 (1994).
5. Chaudhary, J., Johnson, J., Kim, G. & Skinner, M. K. Hormonal regulation and differential actions of the helix-loop-helix transcriptional inhibitors of differentiation (Id1, Id2, Id3, and Id4) in Sertoli cells. *Endocrinology.* **142,** 1727-1736 (2001).
6. Itman, C. & Loveland, K. L. SMAD expression in the testis: an insight into BMP regulation of spermatogenesis. *Dev. Dyn.* **237,** 97-111 (2008).
7. Lin, J. *et al*. Expression of genomic functional estrogen receptor 1 in mouse Sertoli cells. *Reprod. Sci.* **21,** 1411-1422 (2014).
8. Zarkower, D. & Murphy, M. W. DMRT1: An ancient sexual regulator required for human gonadogenesis. *Sex. Dev.* **16**, 112-125 (2022).
9. Buaas, F. W., Val, P. & Swain, A. The transcription co-factor CITED2 functions during sex determination and early gonad development. *Hum. Mol. Genet.* **18,** 2989-3001 (2009).
10. De Gendt, K., Verhoeven, G., Amieux, P. S. & Wilkinson, M. F. Genome-wide identification of AR-regulated genes translated in Sertoli cells in vivo using the RiboTag approach. *Mol. Endocrinol.* **28,** 575-591 (2014).
11. Costa *et al*. Mouse MAELSTROM: the link between meiotic silencing of unsynapsed chromatin and microRNA pathway? *Hum. Mol. Genet.* **15,** 2324-2334 (2006).
12. Cupp, A. S., Kim, G. & Skinner, M. K. Expression and action of transforming growth factor beta (TGFbeta1, TGFbeta2, and TGFbeta3) during embryonic rat testis development. *Biol. Reprod.* **60,** 1304-1313 (1999).
13. Bitgood, M. J., Shen, L. & McMahon, A. P. Sertoli cell signaling by Desert hedgehog regulates the male germline. *Curr. Biol.* **6,** 298-304 (1996).
14. Barakat, B., O'Connor, A. E., Gold, E., de Kretser, D. M. & Loveland, K. L. Inhibin, activin, follistatin and FSH serum levels and testicular production are highly modulated during the first spermatogenic wave in mice. *Reproduction.* **136,** 345-359 (2008).
15. Takase, H. M. & Nusse, R. Paracrine Wnt/β-catenin signaling mediates proliferation of undifferentiated spermatogonia in the adult mouse testis. *Proc. Natl. Acad. Sci. U S A* **113**, E1489-E1497; 10.1073/pnas.1601461113 (2016).
16. Schmahl, J., Kim, Y., Colvin, J. S., Ornitz, D. M. & Capel, B. Fgf9 induces proliferation and nuclear localization of FGFR2 in Sertoli precursors during male sex determination *Development.* **131,** 3627-3636 (2004).
17. Kirschner, K. M., Sciesielski, L. K., Krueger, K. & Scholz, H. Wilms tumor protein-dependent transcription of VEGF receptor 2 and hypoxia regulate expression of the testis-promoting gene Sox9 in murine embryonic gonads. *J. Biol. Chem.* **292,** 20281-20291 (2017).

18. Wang, H. *et al*. Immunoexpression of Tyro 3 family receptors--Tyro 3, Axl, and Mer--and their ligand Gas6 in postnatal developing mouse testis. *J. Histochem. Cytochem.* **53,** 1355-1364 (2005).
19. Perälä, N. M., Immonen, T. & Sariola, H. The expression of plexins during mouse embryogenesis. *Gene Expr. Patterns.* **5,** 355-362 (2005).
20. Gungor-Ordueri, N. E., Tang, E. I., Celik-Ozenci, C. & Cheng, C. Y. Ezrin is an actin binding protein that regulates sertoli cell and spermatid adhesion during spermatogenesis. *Endocrinology.* **155,** 3981-3995 (2014).
21. Kumar, A., Dumasia, K., Deshpande, S., Gaonkar, R. & Balasinor, N. H. Actin related protein complex subunit 1b controls sperm release, barrier integrity and cell division during adult rat spermatogenesis. *Biochim. Biophys. Acta.* **1863,** 1996-2005 (2016).
22. De Gendt, K. *et al*. Expression of Tubb3, a beta-tubulin isotype, is regulated by androgens in mouse and rat Sertoli cells. *Biol. Reprod.* **85,** 934-945 (2011).
23. Neely, M. D. & Boekelheide, K. Sertoli cell processes have axoplasmic features: an ordered microtubule distribution and an abundant high molecular weight microtubule-associated protein (cytoplasmic dynein). *J. Cell Biol.* **107,** 1767-1776 (1988).
24. Zakrzewski, P., Lenartowska, M. & Buss, F. Diverse functions of myosin VI in spermiogenesis. *Histochem. Cell Biol.* **155,** 323-340 (2021).
25. Suetsugu, S. *et al*. Male-specific sterility caused by the loss of CR16. *Genes Cells.* **12,** 721-733 (2007).
26. Zhang, C. *et al*. A chromatin modifier regulates Sertoli cell response to mono-(2-ethylhexyl) phthalate (MEHP) via tissue inhibitor of metalloproteinase 2 (TIMP2) signaling. *Biochim. Biophys. Acta.* **1839,** 1170-1182 (2014).
27. Crépieux, P. *et al*. The ERK-dependent signalling is stage-specifically modulated by FSH, during primary Sertoli cell maturation. *Oncogene.* **20,** 4696-4709 (2001).
28. Li, Y. *et al*. Immunolocalization and regulation of cystatin 12 in mouse testis and epididymis. *Biol. Reprod.* **73,** 872-880 (2005).
29. Liu, Y. *et al*. JMY expression by Sertoli cells contributes to mediating spermatogenesis in mice. *FEBS J.* **287,** 5478-5497 (2020).
30. Lie, P. P., Mruk, D. D., Lee, W. M. & Cheng, C. Y. Epidermal growth factor receptor pathway substrate 8 (Eps8) is a novel regulator of cell adhesion and the blood-testis barrier integrity in the seminiferous epithelium. *FASEB J.* **23,** 2555-2567 (2009).
31. Zheng, M. *et al*. TULP2, a new RNA-binding protein, is required for mouse spermatid differentiation and male fertility. *Front. Cell Dev. Biol.* **9,** 623738; 10.3389/fcell.2021.623738 (2021).
32. Whiley, P. A. F. *et al*. Activin A determines steroid levels and composition in the fetal testis. *Endocrinology.* **161,** bqaa058; 10.1210/endocr/bqaa058 (2020).
33. Beverdam, A. *et al*. Sox9-dependent expression of Gstm6 in Sertoli cells during testis development in mice. *Reproduction.* **137,** 481-486 (2009).
34. Gustin, S. E. *et al*. Testis development, fertility, and survival in Ethanolamine kinase 2-deficient mice. *Endocrinology.* **149,** 6176-6186 (2008).
35. Erickson-Lawrence, M., Zabludoff, S. D. & Wright, W. W. Cyclic protein-2, a secretory product of rat Sertoli cells, is the proenzyme form of cathepsin L. *Mol. Endocrinol.* **5,** 1789-1798 (1991).
36. Siu, M. K., Wong, C. H., Lee, W. M. & Cheng, C. Y. Sertoli-germ cell anchoring junction dynamics in the testis are regulated by an interplay of lipid and protein kinases. *J. Biol. Chem.* **280,** 25029-25047 (2005).

37. Best, D., Sahlender, D. A., Walther, N., Peden, A. A. & Adams, I. R. Sdmg1 is a conserved transmembrane protein associated with germ cell sex determination and germline-soma interactions in mice. *Development.* **135,** 1415-1425 (2008).
38. Bouma, G. J., Affourtit, J. P., Bult, C. J. & Eicher, E. M. Transcriptional profile of mouse pre-granulosa and Sertoli cells isolated from early-differentiated fetal gonads. *Gene Expr. Patterns.* **7,** 113-123 (2007).
39. Holappa, K. *et al*. Primary structure of a sperm cell anion exchanger and its messenger ribonucleic acid expression during spermatogenesis. *Biol. Reprod.* **61,** 981-986 (1999).
40. Huber, R. D. *et al*. Characterization of two splice variants of human organic anion transporting polypeptide 3A1 isolated from human brain. *Am. J. Physiol. Cell Physiol.* **292,** C795-C806 (2007).
41. Adams, A., Yoo, K. M. & Vogl, W. Lipid transfer machinery is present at membrane contact sites associated with the internalization of junctions in Sertoli cells. *Biol. Reprod.* **101,** 662-663 (2019).
42. Cory, A. T., Boyer, A., Pilon, N., Lussier, J. G. & Silversides, D. W. Presumptive pre-Sertoli cells express genes involved in cell proliferation and cell signalling during a critical window in early testis differentiation. *Mol. Reprod. Dev.* **74,** 1491-1504 (2007).
43. Soffientini, U. *et al*. Identification of Sertoli cell-specific transcripts in the mouse testis and the role of FSH and androgen in the control of Sertoli cell activity. *BMC Genomics.* **18,** 972; 10.1186/s12864-017-4357-3 (2017).
44. Law, G. L., McGuinness, M. P., Linder, C. C. & Griswold, M. D. Expression of apolipoprotein E mRNA in the epithelium and interstitium of the testis and the epididymis. *J. Androl.* **18,** 32-42 (1997).
45. Hagenäs, L. *et al*. Sertoli cell origin of testicular androgen-binding protein (ABP). *Mol. Cell. Endocrinol.* **2,** 339-350 (1975).
46. Jin, J. *et al*. Defb19 regulates the migration of germ cell and is involved in male fertility. *Cell Biosci.* **12,** 188; 10.1186/s13578-022-00924-1 (2022).
47. Bailey, R. & Griswold, M. D. Clusterin in the male reproductive system: localization and possible function. *Mol. Cell. Endocrinol.* **151,** 17-23 (1999).
48. Zomer, H. D. & Reddi, P. P. Characterization of rodent Sertoli cell primary cultures. *Mol. Reprod. Dev.* **87,** 857-870 (2020).
49. Zheng, B. *et al*. Quantitative proteomics reveals the essential roles of stromal interaction molecule 1 (STIM1) in the testicular cord formation in mouse testis. *Mol. Cell. Proteomics.* **14,** 2682-2691 (2015).
50. Lyon, K. *et al*. Ca2+ signaling machinery is present at intercellular junctions and structures associated with junction turnover in rat Sertoli cells. *Biol. Reprod.* **96,** 1288-1302 (2017).
51. Yu, W. *et al*. BEX4 upregulation alters Sertoli cell growth properties and protein expression profiles: An explanation for cadmium-induced testicular Sertoli cell injury. *J. Biochem. Mol. Toxicol.* **31,** 10.1002/jbt.21908 (2017).
52. Sarraj, M. A. *et al*. Expression of Wsb2 in the developing and adult mouse testis. *Reproduction.* **133,** 753-761 (2007).
53. Svingen, T., Beverdam, A., Verma, P., Wilhelm, D. & Koopman, P. Aard is specifically up-regulated in Sertoli cells during mouse testis differentiation. *Int. J. Dev. Biol.* **51,** 255-258 (2007).
54. Smith, L. *et al*. Candidate testis-determining gene, Maestro (Mro), encodes a novel HEAT repeat protein. *Dev. Dyn.* **227,** 600-607 (2003).
55. Smith, L. *et al*. The Maestro (Mro) gene is dispensable for normal sexual development and fertility in mice. *PloS One.* **3,** e4091; 10.1371/journal.pone.0004091 (2008).

56. McClelland, K. S. *et al*. Purification and transcriptomic analysis of mouse fetal leydig cells reveals candidate genes for specification of gonadal steroidogenic cells. *Biol. Reprod.* **92,** 1-17 (2015).

Supplementary Table. qRT-PCR primers

Primer name	Forward	Reverse
Aldh1a3	GGGTCACACTGGAGCTAGGA	CTGGCCTCTTCTTGGCGAA
Basp1	GCGAGGCCAAAAAGACTGAG	CCGCGCTGCTAGGTTTAGAG
Bcam	TCAGCGTCGGTCTTTTGCTAC	CAACACTCATCTCCAAAGCCTC
Cited1	AACCTTGGAGTGAAGGATCGC	GTAGGAGAGCCTATTGGAGATGT
Clu	AGCAGGAGGTCTCTGACAATG	GGCTTCCTCTAAACTGTTGAGC
Cst12	CGTGTTCCACTTCAACGAAAAC	GCCCATCTCCAGGTCTACTAAAT
Ctsl	ATCAAACCTTTAGTGCAGAGTGG	CTGTATTCCCCGTTGTGTAGC
Ddx4	CAGTTTGCAATGTGAGCTTTG	GGGGGAAATGTGTTTCATCTT
Defb36	GCGTCTACGTCTACTGCACAA	GCTTCACGCAACACATCTTCC
Dhh	CCCGACATAATCTTCAAGGATGA	GCGATGGCTAGAGCGTTCAC
Dmrt1	GGTTGTAACCAAGTTTTCAGGA	CCGCTCTTCTCACTGGTCA
Emx2	TCAGCTACGCCAATTCCAGTC	ACCAAGTCCGGGTTGGAGTA
Fgfr2	GCCTCTCGAACAGTATTCTCCT	ACAGGGTTCATAAGGCATGGG
Gstm6	ACAGGTCATGGACACTCGAAT	TGGCTTCCGTTTCTCAAAGTC
Hprt	GCAGTACAGCCCCAAAATGG	GGTCCTTTTCACCAGCAAGCT
Hsd3b1	TGGACAAAGTATTCCGACCAGA	GGCACACTTGCTTGAACACAG
Id4	CAGTGCGATATGAACGACTGC	GACTTTCTTGTTGGGCGGGAT
Mro	GCCCGGACCTTACTGGATG	GAGCGAATCTTGGGTCTGGTT
Myh11	GAGCAAACTCAGGAGAGGAAAC	GTCCCGAGCGTCCATTTCTTC
Pax8	AGTCACCCCAGTCGGATTC	CTGTGAGTCGATGCTTAGCCG
Pbx1	ACCTCCTTCGAGAGCAAAGC	GCTGAACTTGCGGTGGATGAT
Pecam1	ACGCTGGTGCTCTATGCAAG	TCAGTTGCTGCCCATTCATCA
Podxl	ACAAGACTAAAACAAGCTCTCCC	GCTGAGAAGAGACGTTGATTAGC
Sema6c	GCCCCAGTCTTCCACTCTTG	CCGGGGTCTCGATGTCCTT
Tbx1	CTGTGGGACGAGTTCAATCAG	TTGTCATCTACGGGCACAAAG
Tbx2	CCGATGACTGCCGCTATAAGT	CCATCCACTGTTCCCCTGT
Tead2	GAAGACGAGAACGCGAAAGC	GATGAGCTGTGCCGAAGACA
Tubb3	AGGTGCGTGAGGAGTACCC	AGGGCTTCATTGTCGATGCAG
